# The association between thyroid and breast cancers: a bidirectional mendelian randomization study

**DOI:** 10.3389/fendo.2023.1185497

**Published:** 2023-10-25

**Authors:** Jinchi Liu, Leifeng Liang

**Affiliations:** ^1^ Department of Oncology, The Sixth Affiliated Hospital of Guangxi Medical University, The First People’s Hospital of Yulin, Guangxi, China; ^2^ China Medical University, Shenyang, China

**Keywords:** breast cancer, thyroid cancer, Mendelian randomization, casual effect, genetics

## Abstract

**Background:**

Thyroid and breast cancers are the two most frequent types of endocrine-related tumors among women worldwide, and their incidence is still on the rise. Observational studies have shown a relationship between thyroid and breast cancers. Nevertheless, many confounders predispose the results to interference. Accordingly, we performed a two-sample Mendelian randomization (MR) study to investigate the causal association between thyroid and breast cancers.

**Methods:**

We acquired breast cancer data from the UK Biobank (13,879 breast cancer cases and 198,523 controls) and the Breast Cancer Association Consortium (BCAC; 122,977 breast cancer cases and 105,974 controls), and thyroid cancer data from FinnGen Biobank (989 thyroid cancer and 217,803 controls). Then, the multiplicative random effects inverse variance weighting (IVW), weight median (WM), and MR Egger methods were executed for MR analysis.

**Results:**

Overall, IVW showed a causal effect of breast cancer on thyroid cancer using the BCAC dataset (odds ratio [OR] = 1.17; 95% confidence interval [CI] = 1.036–1.322; P = 0.011), and this relationship was also supported by the UK Biobank dataset (OR = 23.899; 95% CI = 2.331–245.003; P = 0.007), which showed that breast cancer patients were more likely to be diagnosed with thyroid cancer. On the whole, the reverse MR analysis did not show a causal effect of breast cancer on thyroid cancer. However, IVW showed a causal effect of thyroid cancer on estrogen receptor -negative breast cancer using the BCAC dataset (OR = 1.019; 95% CI = 1.001–1.038; P = 0.043), which suggested that people with thyroid cancer were more likely to develop breast cancer.

**Conclusions:**

Breast cancer represents a possible risk factor for thyroid cancer and thyroid cancer also represents a possible risk factor for ER-negative breast cancer. Future studies using powerful genetic tools to determine the causal relationship between breast and thyroid cancers are required.

## 1 Introduction

Breast cancer is a major contributor to cancer incidence globally, 2.3 million people were newly diagnosed in 2020, accounting for 11.7% of all cancer cases. It is the fifth leading cause of cancer-related deaths worldwide, resulting in 685,000 deaths (1). Current treatment methods, including surgery, radiotherapy, chemotherapy, and targeted therapy have made great progress. However, 20–30% of patients with breast cancer undergo metastases, which causes 90% of the deaths (2).

Owing to the continuous development of diagnostic imaging techniques, the frequency of thyroid cancer screening and diagnosis has increased. This also results from the higher incidence of thyroid cancer due to increased exposure to underlying risk factors, such as obesity, and environmental risk factors, such as iodine levels. According to epidemiological statistics, 586,000 cases of thyroid cancer are reported worldwide, with the 9th highest incidence rate in 2020. The global incidence rate is 10.1 per 100,000 in women, which is three times higher than that in men, and one in every 20 cancers diagnosed in women is thyroid cancer (1). Therefore, the possibility of decreasing thyroid cancer incidence by reducing underlying risk factors has attracted the attention of some scholars.

Several observational studies have reported that thyroid cancer is associated with breast cancer. In some studies, patients with thyroid cancer had a significantly increased risk of breast cancer (3, 4). Similarly, other studies have showed that patients with a history of breast cancer are more likely to have an increased risk of thyroid cancer compared to the general population (5). Genetic and environmental factors, as well as treatment modalities, are suspected to play key roles in the association between these cancers. According to previous reports, the increased rate of this co-occurrence was possibly due to a detection bias following primary tumor diagnosis. Currently, the association between thyroid and breast cancers along with their clinical significance is unclear.

In most cases, the available evidence from observation-based epidemiological findings is prone to confounding and reverse causality biases. In Mendelian randomization (MR) studies, genetic variants have been utilized as proxies or instrumental variables for putative risk factors, which overcame these limitations. As a result of the random assignment of genetic variants, the MR approach eliminates the possibility of confounding. In addition, it can avoid reverse causality bias associated with genetic variants assigned before a disease develops. As a result, MR studies are increasingly being used to measure the effects of interventions on disease risk.

For this study, we used publicly available pooled statistics from a large genome-wide association study (GWAS) with a two-sample MR design to assess the relationship between thyroid and breast cancers.

## 2 Materials and methods

### 2.1 Study design

**Figure 1:**
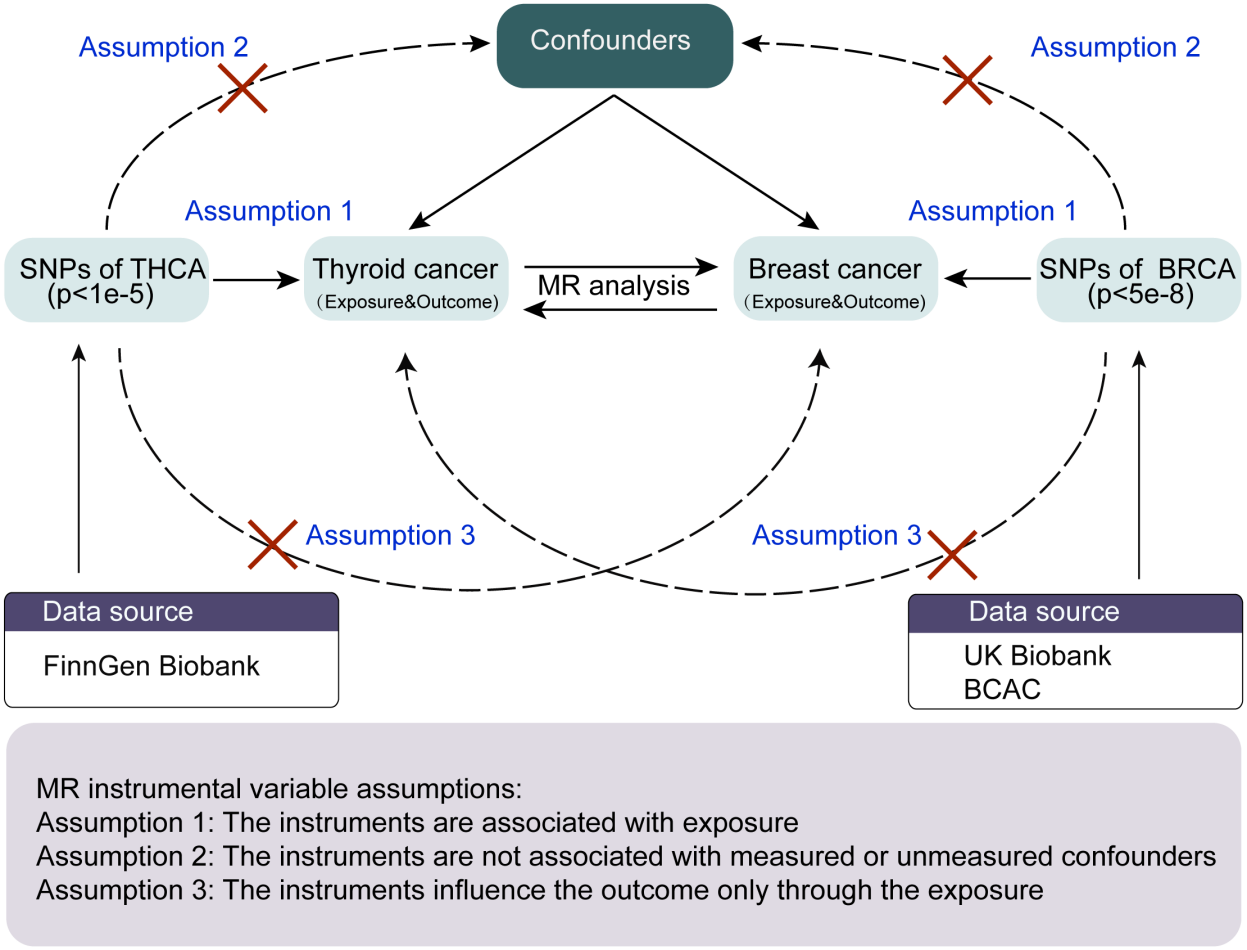
The workflow of the MR analysis in the present study. MR, Mendelian randomization; THCA, thyroid cancer; BRCA, breast cancer; BCAC, breast cancer association consortium.

The overall study design of the two-way, two-sample MR analysis is shown in **Figure 1**. The MR analysis is based on three critical assumptions (6): 1) Genetic variation for Instrumental variables (IVs) must be strongly related to the exposure of interest, the so-called “correlation” hypothesis; 2) genetic variation should not be correlated with any of the confounding factors of expose-outcome associations, the so-called “independence” hypothesis; and 3) the results can only be affected by exposure, which is also referred to as the “exclusion-restriction” hypothesis, i.e., there is no pleiotropy. This two-sample MR study evaluated the effect of thyroid cancer characteristics on breast cancer using summation-level statistics from a GWAS and then assessed the opposite direction (breast cancer versus thyroid cancer characteristics). The study was conducted in compliance with the enhanced observational epidemiological study report-mendelian randomization statement (7).

### 2.2 Data source

The features of the data sources are listed in **Table 1**. All participants in the original GWAS were of European descent. GWAS summary statistics for thyroid cancer (989 thyroid cancer patients and 217,803 controls) were obtained from the FinnGen Consortium. GWAS summary statistics for breast cancer were obtained from the Breast Cancer Association Consortium (BCAC) (8), which included 228,951 European individuals (122,977 breast cancer cases and 105,974 controls) and the UK Biobank (9) (13,879 breast cancer cases and 198,523 controls). As powerful predictors and prognostic markers of breast cancer, estrogen receptors have a significant impact on its occurrence, management, and outcome (10). Therefore, the GWAS summary statistics for breast cancer subtypes according to the estrogen receptor expression were also from the BCAC (8), including estrogen receptor(ER)-positive (69,501 breast cancer cases and 105,974 controls) and ER-negative (21,468 breast cancer cases and 105,974 controls) breast cancers. GWAS summary statistics were collected from the MRC IEU Open GWAS data infrastructure (11, 12), which contains a collection of 214,846,534,918 genetic associations from 39,994 GWAS abstract datasets.

**Table 1:** Description of breast and thyroid cancer sources.

GWAS ID	Phenotype	Cases (n)	Controls (n)	Consortium
ieu-a-1126	Breast cancer	122977	105974	BCAC
ieu-b-4810	Breast cancer	13879	198523	UKBB
ieu-a-1127	ER+ breast cancer	69501	105974	BCAC
ieu-a-1128	ER- breast cancer	21468	105974	BCAC
finn-b-C3_THYROID_GLAND	Thyroid cancer	989	217803	FinnGen

The two sets of Single Nucleotide Polymorphisms associated with breast cancer are independent.

BCAC, Breast Cancer Association Consortium; UKBB, UK biobank; FinnGen, FinnGen Biobank; ER+, Estrogen receptor positive; ER-, Estrogen receptor positive.

### 2.3 Selection of instrumental variables

In order to study the first MR hypothesis, the selected Single Nucleotide Polymorphism (SNPs) must be genome-wide and significantly related to the exposure under interest (P < 5 × 10^-8^). Linkage disequilibrium (LD) clustering was conducted to obtain independent SNPs with the standard (LD R²< 0.001, LD distance > 10,000 kb). Following screening, 142 and 34 SNPs for breast cancer from the UK Biobank and BCAC, respectively were identified as IV candidates. In selecting genome-wide SNPs significantly associated with thyroid cancer, only three SNPs were identified; therefore, we used an optional threshold of implicative correlation (P < 1 × 10^-5^) and consequently selected 20 SNPs. And then we removed SNPs related to outcome variables based on a threshold of 0.05. Last, we further searched the PhenoScaner database to exclude SNPs that may be related to confounding factors. The obtained instrumental variables were all in line with the above screening process.

Further, we excluded exposure-related SNPs that could not be substituted in the resultant dataset. Those that showed a straightforward association with the outcome (P < 0.05) were eliminated to minimize underlying polymorphisms. F-statistics of each exposure were calculated with the formula $\frac{R^{2}\;(N-2)}{\left(1-R^{2}\right)}$ to determine the strength of the connection of instruments (13, 14). All exposures with F > 10 were considered strong, as described in **Table S1**.

### 2.4 Statistical analysis

The inverse variance weighting approach provides the highest accuracy estimation, but may be subjective to ineffective instrumental variables and multivariate impacts. Therefore, with regard to the two cancers and their associations reaching regular significance levels (P < 0.05), three additional sensitivity and multi-effect analyses were performed using the weighted median, MR-Egger, and MR-pleiotropy residuals and outliers (MR-PRESSO) methods to check and rectify likely multivariance. The weighted median method provides a precise estimation if at least 50% of instrumental variables are effective. The MR-Egger regression method detects and adjusts polymorphisms. The MR-PRESSO test detects possible outliers and corrects for the estimates of horizontal polymorphism obtained from the MR-PRESSO analysis. Therefore, in this study, outliers were rejected by correcting the estimates of the horizontal polymorphism obtained by the MR-PRESSO analysis. All statistical analyses were performed in the Windows environment using “TwoSampleMR” (version 0.5.6) and “forestplot” in R 4.1.0. P < 0.05 was considered statistically significant.

## 3 Results

### 3.1 Screening of SNPs

In general, the included GWAS data were published in 2021 and mainly based on European descent (**Table 1**), and the F-statistics of all the exposures were >10, which is the threshold value to distinguish between strong and weak instrumental variables (**Table S1**).

### 3.2 Causal effect of breast cancer on thyroid cancer

**Figure 2:**
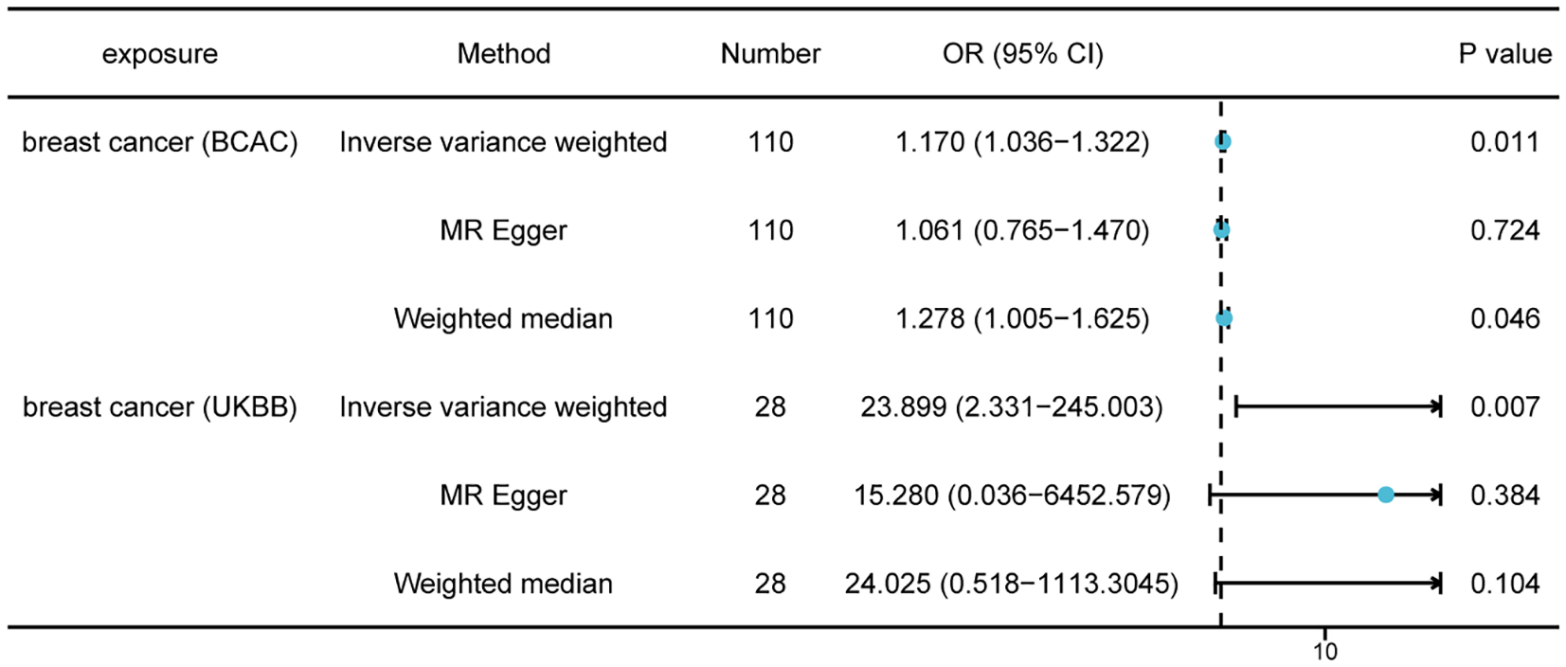
Mendelian randomization results of causal effects of breast cancer on thyroid cancer (P < 5 × 10^−8^). Number, number of SNPs included in the analysis; OR, odds ratio; CI, confidence interval.

First, we selected 125 SNPs associated with breast cancer from the BCAC datasets as valid IVs. Following a critical screening process, 110 SNPs related to breast cancer were selected as effective IVs for thyroid cancer. Causal correlation analysis of breast and thyroid cancers used the multiplicative random effects inverse variance weighting (IVW), weighted median (WM), and MR-Egger methods. The results from the IVW method showed a causal relationship between breast and thyroid cancers (odds ratio [OR] = 1.17; 95% confidence interval [CI] = 1.036–1.322; P = 0.011), which indicated that patients with breast cancer were more likely to be diagnosed with thyroid cancer (**Figure 2**). The results from the WM method also supported the causal effect of breast cancer on thyroid cancer (OR = 1.278; 95% CI = 1.005–1.625; P = 0.046). Egger intercepts suggested no evidence of pleiotropy (P = 0.820). We also selected 28 SNPs associated with breast cancer from the UK Biobank for validation. The results from the IVW method in the validation datasets also showed a causal relationship between breast and thyroid cancers (OR = 23.899; 95% CI = 2.331–245.003; P = 0.007), which validated our finding in BCAC dataset (**Figure 2**). The Egger intercept showed no evidence of polymorphism (P = 0.873). The absence of SNPs dominated the MR estimates to a large extent, as shown in the “leave-one-out test.” Overall, funnel, scatter, and forest plots showed variant-specific causal estimates, suggesting no significant heterogeneity among SNPs for breast and thyroid cancers (**Figures S1**, **S2**).

### 3.3 Causal effect of thyroid cancer on breast cancer

**Figure 3:**
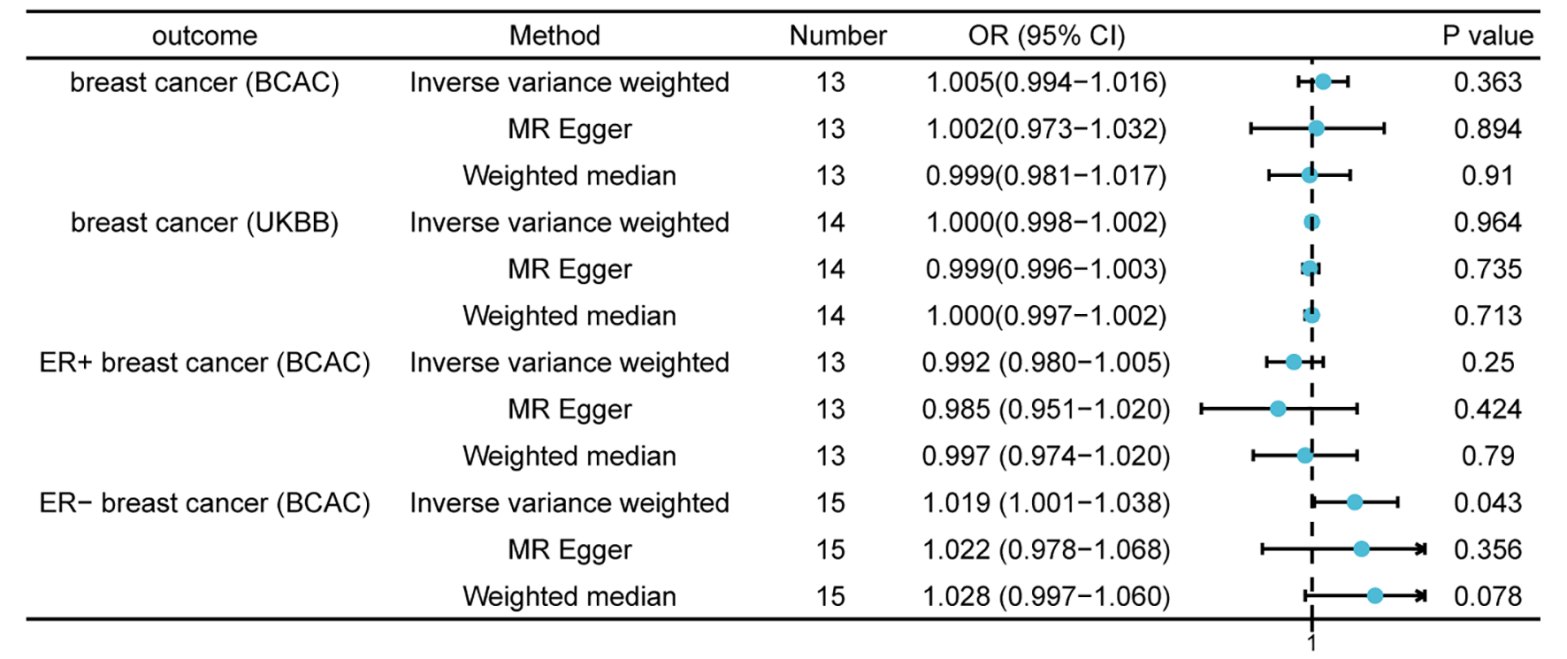
Mendelian randomization results of causal effects of thyroid cancer on breast cancer (P < 1 × 10^−5^). Number, number of SNPs included in the analysis; OR, odds ratio; CI, confidence interval.

Following a critical screening process, 13 and 14 SNPs related to thyroid cancer in the BCAC and UK Biobank, respectively were selected as valid IVs for breast cancer. Causal effect analysis of thyroid cancer on breast cancer also used the multiplicative random effects IVW, WM, and MR-Egger methods. However, there was no evidence suggesting the causal effects of thyroid cancer on breast cancer using various MR methods in both the BCAC(OR = 1.005; 95% CI = 0.994- 1.016; P = 0.363) and UK Biobank datasets(OR = 1.000; 95% CI = 0.998–1.002; P = 0.964). A in the BCAC database, patients with breast cancer were divided into ER-positive and ER-negative subgroups according to ER expression in patients with breast cancer. Although a causal effect of thyroid cancer on ER-positive breast cancer was not observed, the IVW method showed a causal relationship between thyroid cancer and ER-negative breast cancer (OR = 1.019; 95% CI = 1.001–1.038; P = 0.043), which indicated that patients with thyroid cancer had an increased risk of ER-negative breast cancer (**Figure 3**). The absence of SNPs dominated the MR estimates to a large extent, as shown in the “leave-one-out test.” Overall, funnel, scatter, and forest plots showed variant-specific causal estimates, suggesting no significant heterogeneity among SNPs for breast and thyroid cancers (**Figures S3**-**S6**).

## 4 Discussion

To the best of our knowledge, this was the first two-sample MR analysis to investigate the bidirectional association between thyroid and breast cancers. We identified genetic susceptibility to breast cancer that was strongly correlated with an enhanced risk of thyroid cancer using summary data from a previous GWAS. The relationship between the two cancers was also validated using validation datasets. Although the results of the reverse MR analysis did not indicate causal effects of thyroid cancer on breast cancer in general, through subgroup analysis, we found that thyroid cancer was associated with a higher risk of ER-negative breast cancer.

Thyroid cancer is one of the most prevalent endocrine malignancies, and it is ranked the fifth most prevalent cancer in women. In 2020, thyroid cancer had the ninth-highest incidence worldwide, and incidence rates continue to rise (15, 16). Multiple primary tumors are responsible for 13.1% and 13.7% of cancer cases in men and women, respectively, and the likelihood of developing a secondary cancer is twice as high for all cancer survivors than for individuals without cancer. Multiple malignancies may co-occur in isolation and at random or may be associated with risk factors such as environmental or inherited susceptibility and therapeutically relevant effects (17). Ron et al. found an increased risk of developing thyroid cancer following breast cancer (Standardized incidence ratio(SIR) = 1.68) and breast cancer after thyroid cancer (SIR=1.89) (18). Hsu et al. reported a combined incidence of 1.59% for breast and thyroid cancers among women, and the correlation was moderately strong (17).

As hormonally reactive tissues, the mammary and thyroid glands share similar endocrine signals. Mammary cells respond to signals from thyroid hormones and can be affected by changes in the thyroid hormone levels. Similarly, thyroid cells respond to sex hormones, especially estrogen, and may undergo carcinogenic transformation upon estrogen stimulation. Both thyroid and sex hormones also show important levels of transcriptional cross-talk, influencing tumorigenesis and sensitization to treatment (19).

Additionally, studies have explored the effects of thyroid cancer treatment with radioactive iodine (RAI) on breast cancer. In a meta-analysis, 14 studies with 200,247 differentiated thyroid cancer(DTC) individuals were included (98,368 treated with RAI and 101,879 not treated with RAI), which indicated that DTC patients receiving RAI did not have a higher risk of developing primary breast cancer than those who were not treated with RAI. These results indicated that RAI therapy did not affect the probability of developing breast cancer (20). A cohort study involving 55,318 women indicated that women who had RT had a similar probability of developing thyroid cancer to those who did not had RT (adjusted HR (aHR) = 1.28, 95% CI = 0.90–1.83) (21).

These results of these studies support the those of the present study. SNPs used as IVs are unaffected by numerous confounding factors, making the MR technique particularly advantageous. Additionally, the sequential order used in causal inference supports the validity of the conclusions.

As previously described, causal estimations are valid when the three assumptions of the MR model are satisfied. In the first assumption, independent SNPs that closely related to the breast and thyroid cancer were selected. In addition, the F-statistic was >10 for each exposure, demonstrating that the selected SNPs were reliable for IV. In addition, the data in this study were obtained from European populations, thus avoiding bias caused by different populations to a certain extent. Second, we used the MR-Egger regression method to assess the bias created by polymorphism in MR and found that the intercept was close to 0 (P > 0.05), showing that there was no polymorphism caused by unknown factors. In addition, no polymorphisms were observed using the MR-PRESSO method. Third, the results of the heterogeneity test confirmed the absence of heterogeneity. In summary, the selected IVs were reasonable, and the study results were convincing. Our research shows that patients with breast cancer are more likely to develop thyroid cancer, and that patients with thyroid cancer have an increased risk of developing ER-negative breast cancer. Therefore, for patients with breast cancer, more emphasis should be placed on preventing thyroid cancer. Similarly, patients with thyroid cancer should be cautious of developing breast cancer by seeking early diagnosis and treatment for a good prognosis.

However, our study had some limitations. First, the study analyzed data from a European population to ensure that the results were not skewed; however, there are limitations to extrapolating our results to other populations. Second, we screened and examined risk factors that were potentially modifiable and reported in previous studies. Those that had not been reported might also influence the pathogenesis of the resulting tumor. Third, the MR method could only be used to analyze cause and effect relationships; the mechanisms behind the increased risk of cancer could not be studied. More comprehensive studies are required to further explore the mechanisms that influence the increased risk for each type of cancer.

## Data availability statement

The datasets presented in this study can be found in online repositories. The original contributions presented in the study are included in the article/**Supplementary Material**. Further inquiries can be directed to the corresponding author.

## Author contributions

LL: conception, design, acquisition, interpretation of data, writing—original draft, and writing—review and editing. JL: data acquisition, analysis, interpretation of data, and writing—original draft. All authors contributed to the article and approved the submitted version.

## References

[1] Sung H Ferlay J Siegel RL Laversanne M Soerjomataram I Jemal A. Global cancer statistics 2020: GLOBOCAN estimates of incidence and mortality worldwide for 36 cancers in 185 countries. CA Cancer J Clin (2021) 71(3):209–49. doi: 10.3322/caac.21660 33538338

[2] Britt KL Cuzick J Phillips KA. Key steps for effective breast cancer prevention. Nat Rev Cancer (2020) 20(8):417–36. doi: 10.1038/s41568-020-0266-x 32528185

[3] Brown AP Chen J Hitchcock YJ Szabo A Shrieve DC Tward JD. The risk of second primary Malignancies up to three decades after the treatment of differentiated thyroid cancer. J Clin Endocrinol Metab (2008) 93(2):504–15. doi: 10.1210/jc.2007-1154 18029468

[4] Consorti F Di Tanna G Milazzo F Antonaci A. Nulliparity enhances the risk of second primary Malignancy of the breast in a cohort of women treated for thyroid cancer. World J Surg Oncol (2011) 9:88. doi: 10.1186/1477-7819-9-88 21835042 PMC3174118

[5] Evans HS Lewis CM Robinson D Bell CM Møller H Hodgson SV. Incidence of multiple primary cancers in a cohort of women diagnosed with breast cancer in southeast England. Br J Cancer (2001) 84(3):435–40. doi: 10.1054/bjoc.2000.1603 PMC2363744 11161413

[6] Lawlor DA. Commentary: Two-sample Mendelian randomization: opportunities and challenges. Int J Epidemiol (2016) 45(3):908–15. doi: 10.1093/ije/dyw127 PMC5005949 27427429

[7] Skrivankova VW Richmond RC Woolf BAR Yarmolinsky J Davies NM Swanson SA. Strengthening the reporting of observational studies in epidemiology using mendelian randomization: the STROBE-MR statement. JAMA (2021) 326(16):1614–21. doi: 10.1001/jama.2021.18236 34698778

[8] Michailidou K Lindström S Dennis J Beesley J Hui S Kar S. Association analysis identifies 65 new breast cancer risk loci. Nature (2017) 551(7678):92–4. doi: 10.1038/nature24284 PMC5798588 29059683

[9] Sudlow C Gallacher J Allen N Beral V Burton P Danesh J. UK biobank: an open access resource for identifying the causes of a wide range of complex diseases of middle and old age. PloS Med (2015) 12(3):e1001779. doi: 10.1371/journal.pmed.1001779 25826379 PMC4380465

[10] Sommer S Fuqua SA. Estrogen receptor and breast cancer. Semin Cancer Biol (2001) 11(5):339–52. doi: 10.1006/scbi.2001.0389 11562176

[11] Hemani G Zheng J Elsworth B Wade KH Haberland V Baird D. The MR-Base platform supports systematic causal inference across the human phenome. Elife (2018) 7. doi: 10.7554/eLife.34408 PMC5976434 29846171

[12] Elsworth B Zheng H Shi YZ Liang JT Lu LL Chen M. The MRC IEU OpenGWAS data infrastructure. bioRxiv (2020) 14:2020.08.10.244293. doi: 10.1101/2020.08.10.244293

[13] Papadimitriou N Dimou N Tsilidis KK Banbury B Martin RM Lewis SJ. Physical activity and risks of breast and colorectal cancer: a Mendelian randomisation analysis. Nat Commun (2020) 11(1):597. doi: 10.1038/s41467-020-14389-8 32001714 PMC6992637

[14] Wu P Zhang X Zhou P Zhang W Li D Lv M. Assessment of bidirectional relationships between polycystic ovary syndrome and periodontitis: insights from a mendelian randomization analysis. Front Genet (2021) 12:644101. doi: 10.3389/fgene.2021.644101 33868379 PMC8044848

[15] Siegel R Ma J Zou Z Jemal A. Cancer statistics, 2014. CA Cancer J Clin (2014) 64(1):9–29. doi: 10.3322/caac.21208 24399786

[16] Wang E Karedan T Perez CA. New insights in the treatment of radioiodine refractory differentiated thyroid carcinomas: to lenvatinib and beyond. Anticancer Drugs (2015) 26(7):689–97. doi: 10.1097/CAD.0000000000000247 25974026

[17] Hsu CH Huang CL Hsu YH Iqbal U Nguyen PA Jian WS. Co-occurrence of second primary Malignancy in patients with thyroid cancer. Qjm (2014) 107(8):643–8. doi: 10.1093/qjmed/hcu051 24623860

[18] Ron E Curtis R Hoffman DA Flannery JT. Multiple primary breast and thyroid cancer. Br J Cancer (1984) 49(1):87–92. doi: 10.1038/bjc.1984.13 6691901 PMC1976676

[19] Halada S Casado-Medrano V Baran JA Lee J Chinmay P Bauer AJ. Hormonal crosstalk between thyroid and breast cancer. Endocrinology (2022) 163(7). doi: 10.1210/endocr/bqac075 PMC9653009 35587175

[20] Nappi C Klain M Cantoni V Green R Piscopo L Volpe F. Risk of primary breast cancer in patients with differentiated thyroid cancer undergoing radioactive iodine therapy: a systematic review and meta-analysis. Eur J Nucl Med Mol Imaging (2022) 49(5):1630–9. doi: 10.1007/s00259-021-05625-4 34820683

[21] Lin CY Lin CL Huang WS Kao CH. Risk of breast cancer in patients with thyroid cancer receiving or not receiving 131I treatment: A nationwide population-based cohort study. J Nucl Med (2016) 57(5):685–90. doi: 10.2967/jnumed.115.164830 26719377

