# Supplementary material for: The association between thyroid and breast cancers: a bidirectional mendelian randomization study
Source: Front Endocrinol (Lausanne). 2023 Oct 25;14:1185497. doi: 10.3389/fendo.2023.1185497 (PMC10634417; doi:10.3389/fendo.2023.1185497)
Supplement: Supplementary file 1 [file DataSheet_1.docx]

Supplementary Material

## Supplementary Figures


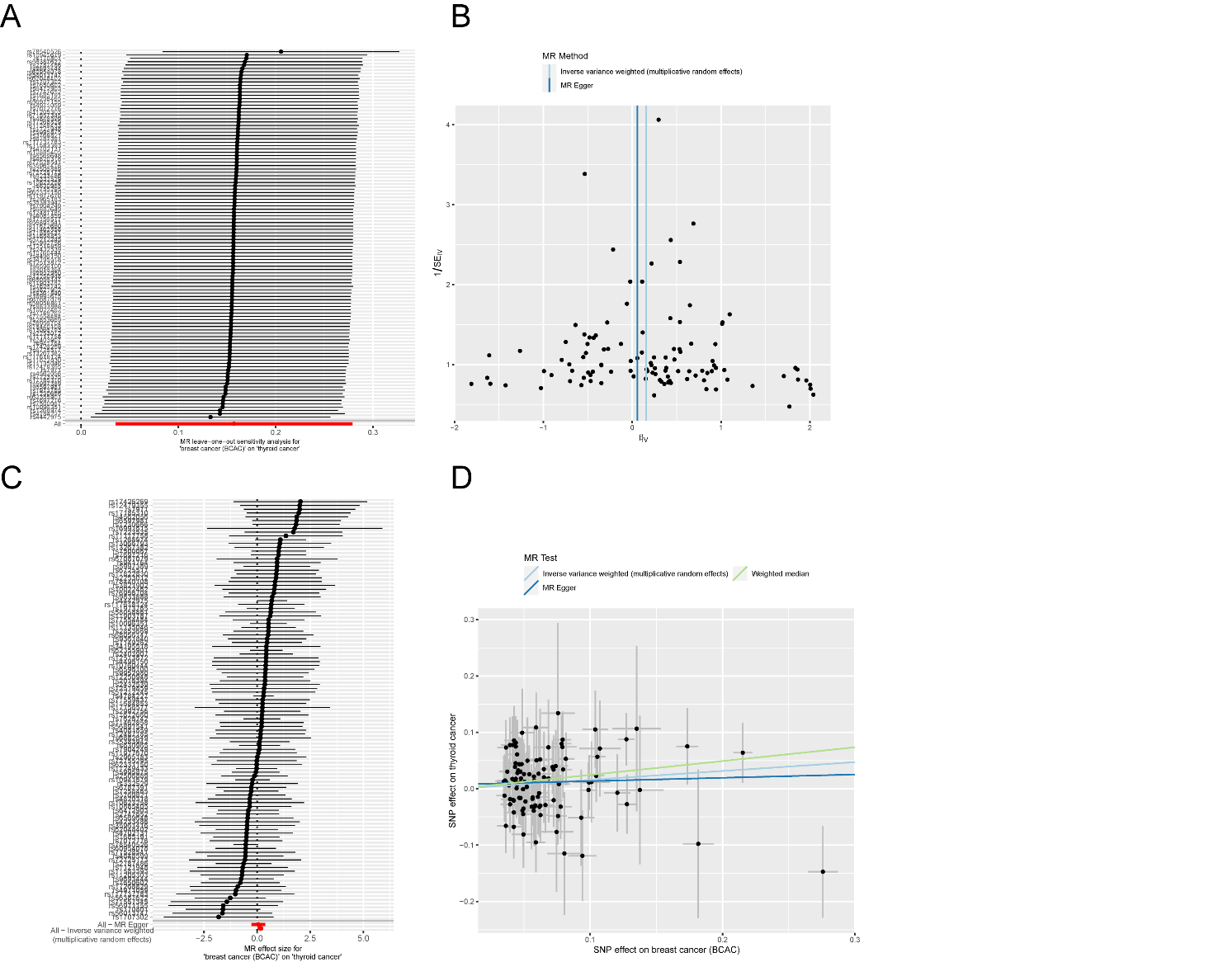


**Supplemental Fig. 1.** Sensitivity analysis of MR estimates on the casual effects of breast cancer in Breast Cancer Association Consortium on thyroid cancer. A, “Leave one out test”; The effect estimates is re-calculated after removal of each SNP to identify if a single SNP is driving the association; B, Funnel plot; the estimates are plotted against the precision of the estimates to test for potential asymmetry. C, Forest plot; It shows the effect estimate of each SNP with 95% confidence interval. D, Scatter plot; SNP-outcome associations are plotted against the SNP-exposure associations to provide the effect estimate for each individual variant. The lines with different colors represent the regression slope fitted by different MR methods.


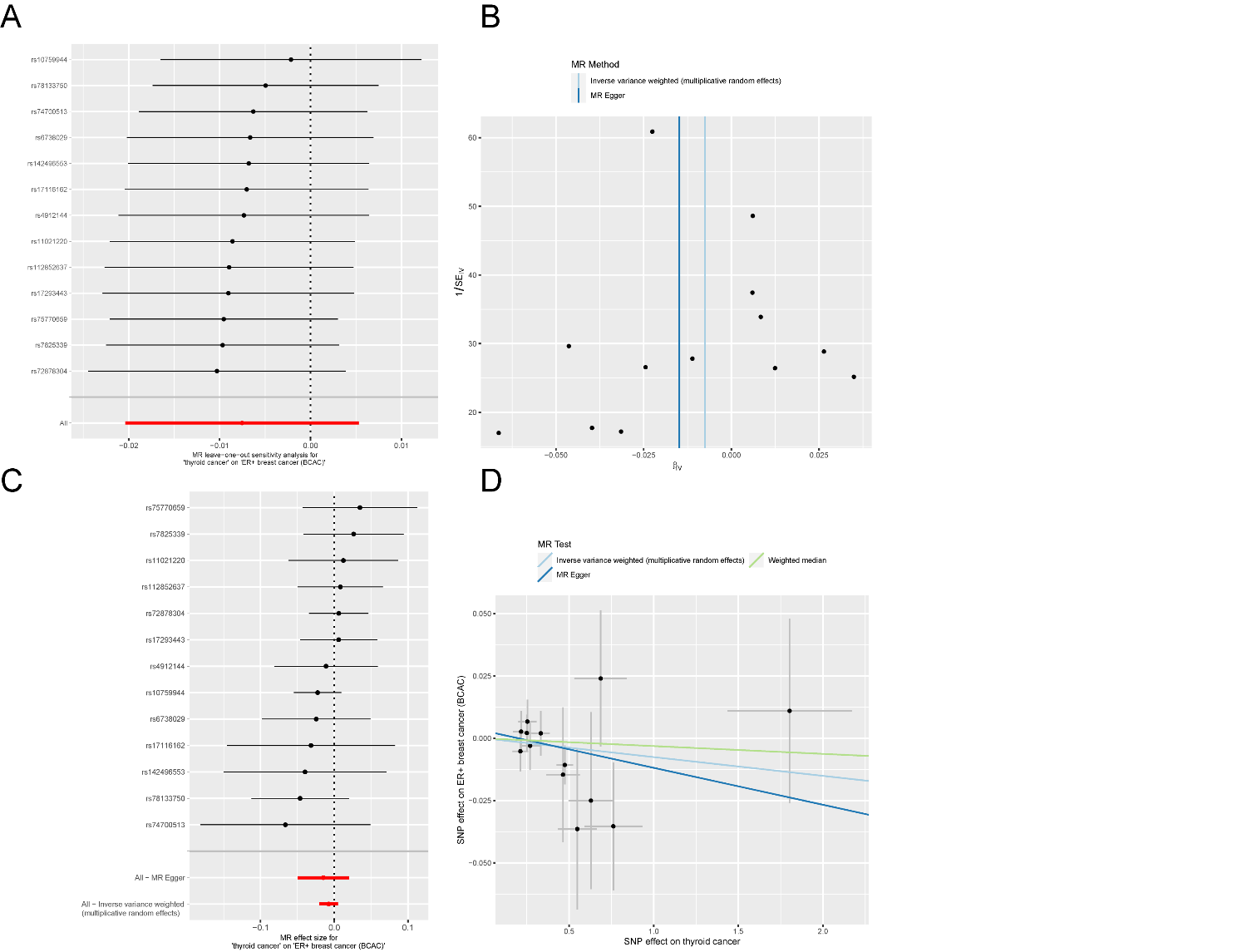


**Supplemental Fig. 2.** Sensitivity analysis of MR estimates on the casual effects of breast cancer in UK Biobank on thyroid cancer. A, “Leave one out test”; The effect estimates is re-calculated after removal of each SNP to identify if a single SNP is driving the association; B, Funnel plot; the estimates are plotted against the precision of the estimates to test for potential asymmetry. C, Forest plot; It shows the effect estimate of each SNP with 95% confidence interval. D, Scatter plot; SNP-outcome associations are plotted against the SNP-exposure associations to provide the effect estimate for each individual variant. The lines with different colors represent the regression slope fitted by different MR methods.


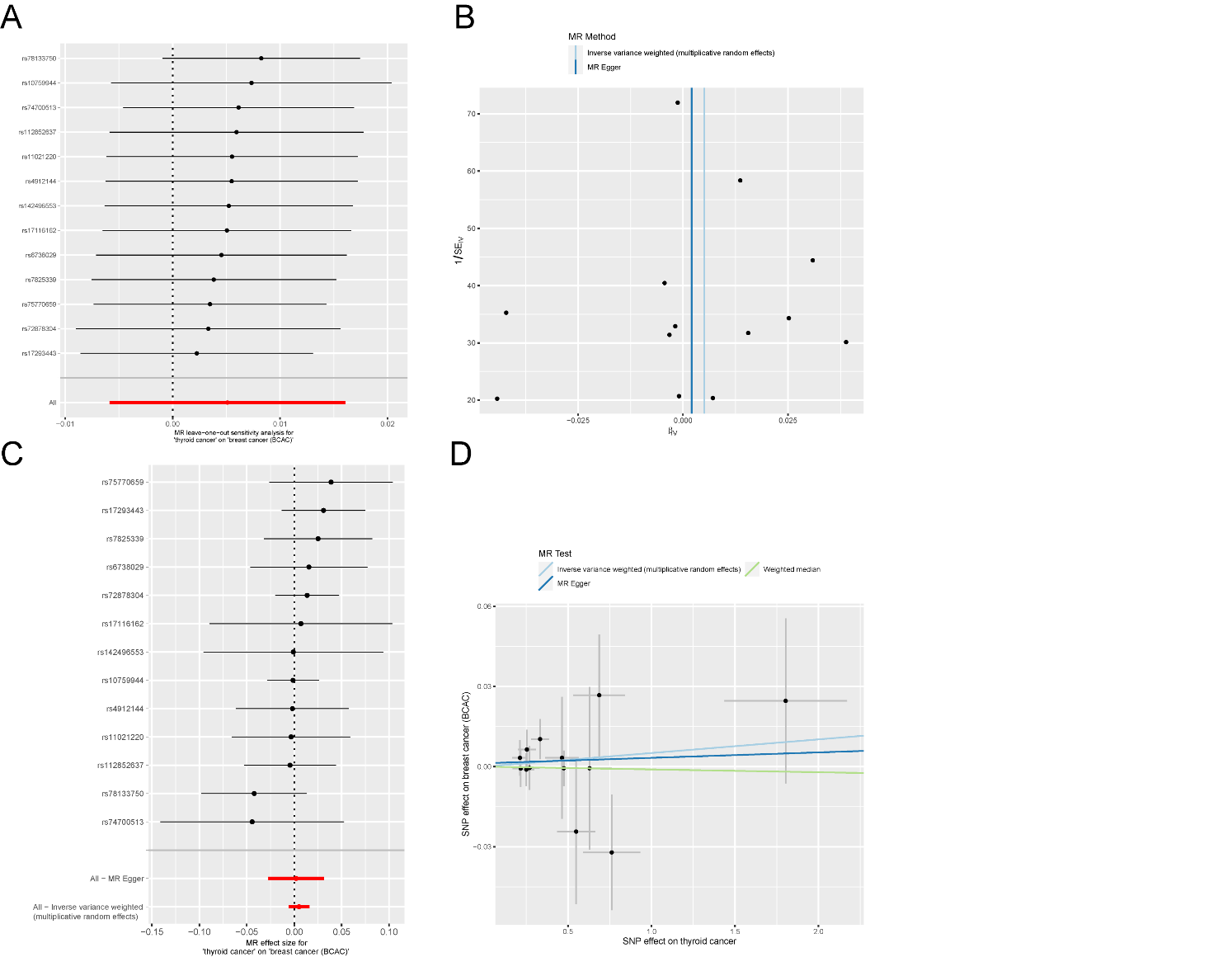


**Supplemental Fig. 3.** Sensitivity analysis of MR estimates on the casual effects of thyroid cancer on breast cancer in Breast Cancer Association Consortium. A, “Leave one out test”; The effect estimates is re-calculated after removal of each SNP to identify if a single SNP is driving the association; B, Funnel plot; the estimates are plotted against the precision of the estimates to test for potential asymmetry. C, Forest plot; It shows the effect estimate of each SNP with 95% confidence interval. D, Scatter plot; SNP-outcome associations are plotted against the SNP-exposure associations to provide the effect estimate for each individual variant. The lines with different colors represent the regression slope fitted by different MR methods.


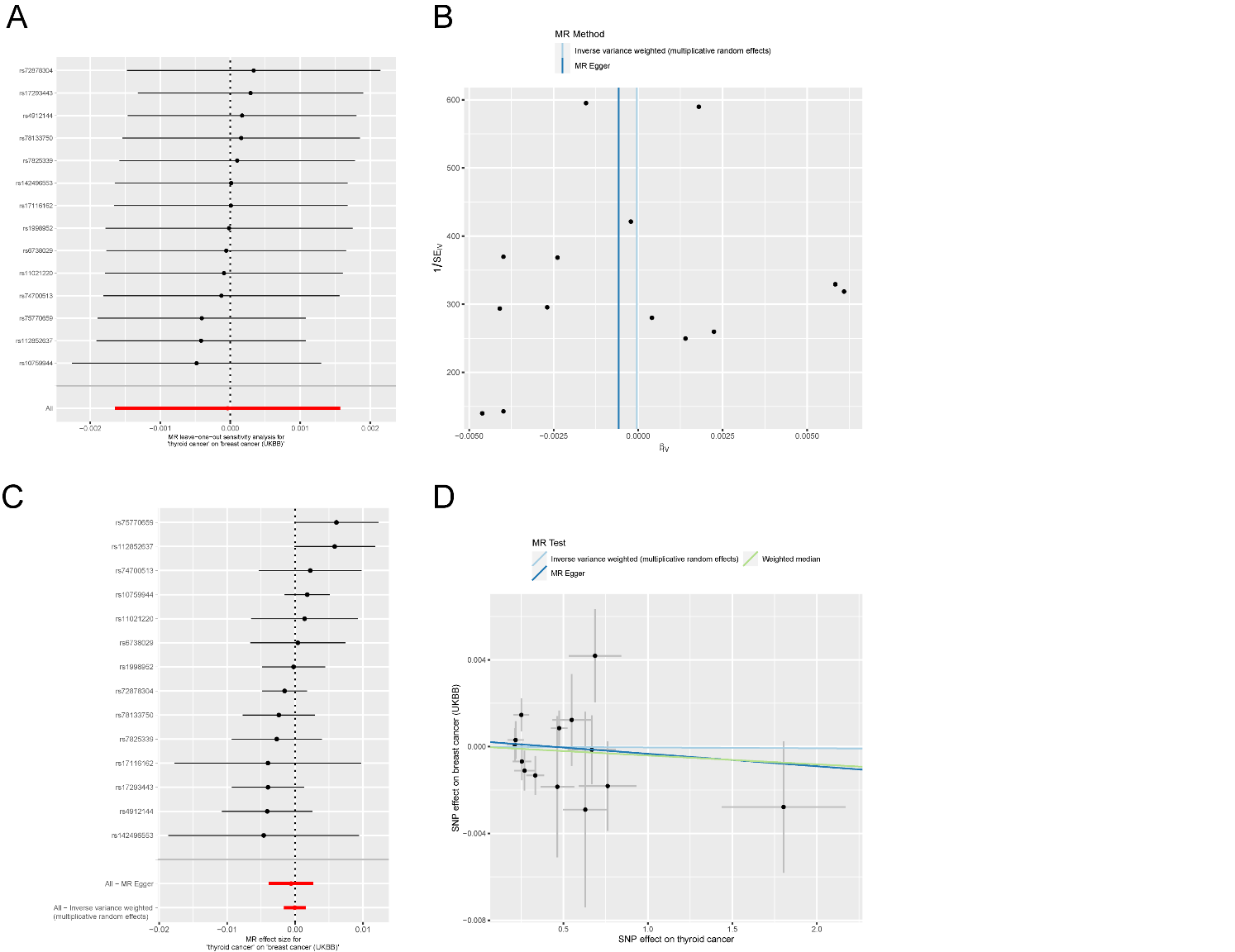


**Supplemental Fig. 4.** Sensitivity analysis of MR estimates on the casual effects of thyroid cancer on breast cancer in UK Biobank. A, “Leave one out test”; The effect estimates is re-calculated after removal of each SNP to identify if a single SNP is driving the association; B, Funnel plot; the estimates are plotted against the precision of the estimates to test for potential asymmetry. C, Forest plot; It shows the effect estimate of each SNP with 95% confidence interval. D, Scatter plot; SNP-outcome associations are plotted against the SNP-exposure associations to provide the effect estimate for each individual variant. The lines with different colors represent the regression slope fitted by different MR methods.


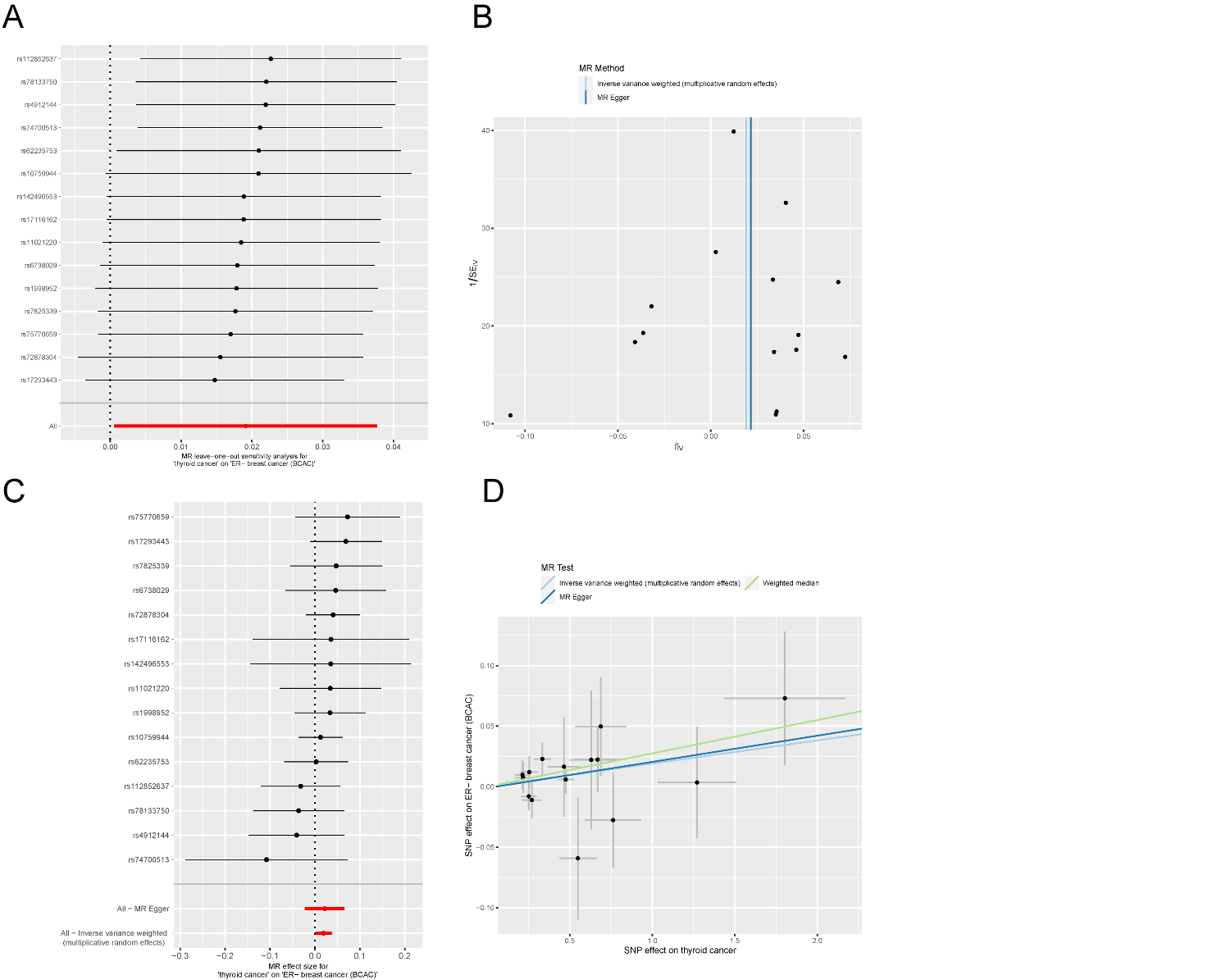


**Supplemental Fig. 5.** Sensitivity analysis of MR estimates on the casual effects of thyroid cancer on estrogen negative breast cancer in Breast Cancer Association Consortium. A, “Leave one out test”; The effect estimates is re-calculated after removal of each SNP to identify if a single SNP is driving the association; B, Funnel plot; the estimates are plotted against the precision of the estimates to test for potential asymmetry. C, Forest plot; It shows the effect estimate of each SNP with 95% confidence interval. D, Scatter plot; SNP-outcome associations are plotted against the SNP-exposure associations to provide the effect estimate for each individual variant. The lines with different colors represent the regression slope fitted by different MR methods.


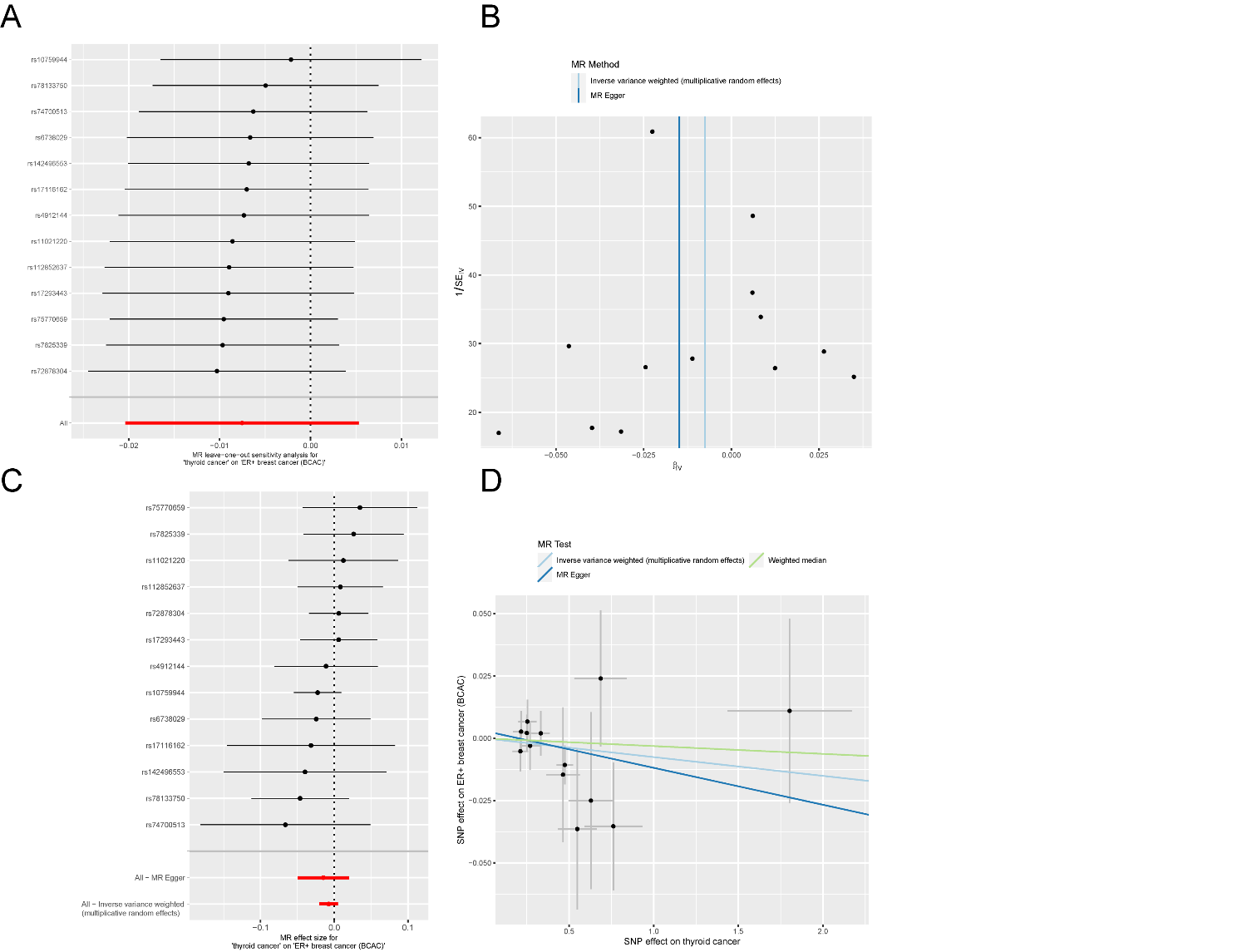


**Supplemental Fig. 6.** Sensitivity analysis of MR estimates on the casual effects of thyroid cancer on estrogen positive breast cancer in Breast Cancer Association Consortium. A, “Leave one out test”; The effect estimates is re-calculated after removal of each SNP to identify if a single SNP is driving the association; B, Funnel plot; the estimates are plotted against the precision of the estimates to test for potential asymmetry. C, Forest plot; It shows the effect estimate of each SNP with 95% confidence interval. D, Scatter plot; SNP-outcome associations are plotted against the SNP-exposure associations to provide the effect estimate for each individual variant. The lines with different colors represent the regression slope fitted by different MR methods.

**Supplemental tables**

**Supplemental Table 1. F statistics of each exposure**

| variable | R_square | F_value |
| --- | --- | --- |
| thyroid cancer to breast cancer (BCAC) | 0.002093 | 26.99101 |
| thyroid cancer to breast cancer (UKBB) | 0.002099 | 27.0644 |
| thyroid cancer to ER+ breast cancer (BCAC) | 0.002093 | 26.99101 |
| thyroid cancer to ER- breast cancer (BCAC) | 0.002323 | 26.80871 |
| breast cancer (BCAC) to thyroid cancer | 0.04681 | 90.62383 |
| breast cancer (UKBB) to thyroid cancer | 0.010052 | 71.87742 |

BCAC, Breast Cancer Association Consortium; UKBB, UK Biobank; ER+, estrogen receptor positive; ER-, estrogen receptor negative.
